# Artificial-Intelligence-Enhanced Mobile System for Cardiovascular Health Management

**DOI:** 10.3390/s21030773

**Published:** 2021-01-24

**Authors:** Zhaoji Fu, Shenda Hong, Rui Zhang, Shaofu Du

**Affiliations:** 1 School of Management, University of Science and Technology of China, Hefei 230026, China; fuzj@mail.ustc.edu.cn (Z.F.); sdu@ustc.edu.cn (S.D.); 2 HeartVoice Medical Technology, Hefei 230027, China; 3 National Institute of Health Data Science at Peking University, Peking University, Beijing 100191, China; 4 Institute of Medical Technology, Health Science Center of Peking University, Beijing 100191, China; 5 Division of Life Science and Medicine, University of Science and Technology of China, Hefei 230026, China; rae@ustc.edu.cn

**Keywords:** electrocardiogram, health management, cardiovascular disease, mobile system, artificial intelligence, deep learning

## Abstract

The number of patients with cardiovascular diseases is rapidly increasing in the world. The workload of existing clinicians is consequently increasing. However, the number of cardiovascular clinicians is declining. In this paper, we aim to design a mobile and automatic system to improve the abilities of patients’ cardiovascular health management while also reducing clinicians’ workload. Our system includes both hardware and cloud software devices based on recent advances in Internet of Things (IoT) and Artificial Intelligence (AI) technologies. A small hardware device was designed to collect high-quality Electrocardiogram (ECG) data from the human body. A novel deep-learning-based cloud service was developed and deployed to achieve automatic and accurate cardiovascular disease detection. Twenty types of diagnostic items including sinus rhythm, tachyarrhythmia, and bradyarrhythmia are supported. Experimental results show the effectiveness of our system. Our hardware device can guarantee high-quality ECG data by removing high-/low-frequency distortion and reverse lead detection with 0.9011 Area Under the Receiver Operating Characteristic Curve (ROC–AUC) score. Our deep-learning-based cloud service supports 20 types of diagnostic items, 17 of them have more than 0.98 ROC–AUC score. For a real world application, the system has been used by around 20,000 users in twenty provinces throughout China. As a consequence, using this service, we could achieve both active and passive health management through a lightweight mobile application on the WeChat Mini Program platform. We believe that it can have a broader impact on cardiovascular health management in the world.

## 1. Introduction

With the rapid development of the worldwide economy and the significant improvement in living standards, peoples’ average life expectancy has increased, which has led to substantial changes in the types of diseases in the population. For example, in China, cardiovascular diseases have become the leading cause of death, and the prevalence of such diseases in China continues to rise. According to the summary of the 2018 Report on Cardiovascular Diseases in China [1], the number of patients with cardiovascular diseases is 290 million.

With the development of health informatics [2,3], clinical data—including those obtained from electrocardiograms (ECGs), electroencephalograms (EEGs), magnetic resonance imaging, computerized tomography scans, and electronic health records (EHRs)—are digitized and stored [4,5] in a Health Informatics System. Among them, ECGs are the primary diagnostic tool for cardiovascular disease both inside and outside hospitals. Common ECG abnormalities such as atrial fibrillation and ventricular arrhythmia are associated with significant mortality and morbidity through the association of risk of death, stroke, hospitalization, heart failure, and coronary artery disease, among others [6,7,8,9,10]. An ECG can be used for diagnosing a wide range of heart diseases, including cardiac arrhythmia and acute coronary syndrome [11,12]. Similar diagnostic measurements, including body temperature, blood pressure, and blood glucose levels, cannot provide the same ability as an ECG to monitor heart health. In addition, unlike a blood glucose monitor, an ECG monitor is noninvasive. Thus, the introduction of an ECG into cardiovascular health management is promising.

Thus far, however, only a small number of successful cardiovascular health management applications based on an ECG have been developed. To understand the associated difficulties, three key challenges are summarized in this paper.

- An ECG is difficult to acquire outside a hospital. In addition, ECG data are easily distorted by environmental noise, other electrical interference, or even irrelevant electrical human physiology activities. Meanwhile, high-quality ECG recorders are extremely large to be carried on a daily basis. If an ECG is noisy, it would only provide insufficient information as to a photoplethysmogram (PPG), which is an optically obtained plethysmogram that can be collected from a Mi Band or Apple Watch (before series 4). Although a PPG can detect rhythm-level abnormalities such as Atrial Fibrillation (AF), it cannot provide useful information regarding heartbeat level abnormalities [13] such as Premature Ventricular Contraction (PVC).
- Most existing ECG monitors can only record data and do not have sufficient analytical capability. Hence, a vast majority of ECG data remain stored in databases that are never used again [14]. Existing diagnostic pattern recognition methods are quite complicated and require significant effort for their development. Hence, only a few institutions use such diagnostic functions in low-priced devices for use outside a hospital setting.
- ECG diagnostic results are difficult to understand by ordinary users. Although there are a few cloud services that can provide analysis capabilities [15], the results include professional terminology, requiring a professional to interpret. Such terms are quite difficult to understand by typical users, thus, it is difficult to encourage ordinary people to use such tools for cardiovascular health management. Such devices require further development to make them acceptable to ordinary users.

To tackle the above challenges, in this paper, we propose an AI-enhanced mobile system for cardiovascular health management for use outside hospitals. Using this system, anyone can easily manage their cardiovascular health. Specifically, our system has the following unique characteristics:

- We developed a mobile system with high-quality data acquisition. Our system consists of hardware devices and a cloud service. The hardware devices are sufficiently lightweight and can be carried throughout the day. Cloud services can be accessed anywhere using the Internet. Data quality is guaranteed by both the hardware design and software algorithms.
- We improved the mobile system using advanced AI algorithms to provide accurate ECG diagnostic results. Our cardiovascular health management system can provide abundant diagnostic reports for ordinary people and doctors. We also deploy it as a cloud service. The entire medical industry can be made more efficient by equipping their ECGs with our cloud service.
- The user interface of the system application is very easy possible. In detail, we built a lightweight mobile app based on the WeChat Mini Program, allowing users to learn how to use the system with little expense. In addition, we created a new heart health score to quantitatively understand the ECG diagnostic results in a similar way to how we interpret body temperature or blood pressure.

Thus far, our system has been used by around 20,000 users from twenty provinces in China. It can also have a broader impact on cardiovascular health management in the world.

## 2. Methods

### 2.1. Overview

**Figure 1:**
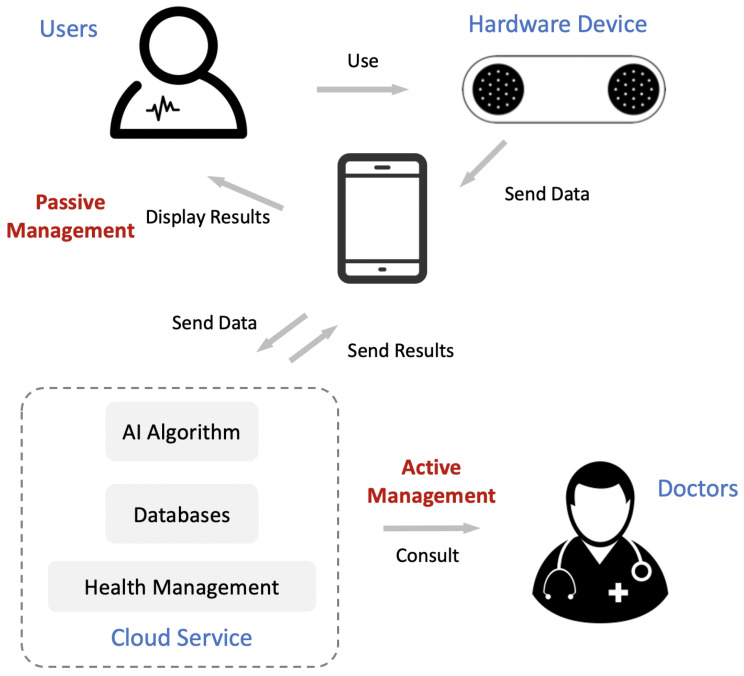
Framework of our mobile cardiovascular health management system. It consists of four critical components: users, hardware device, cloud service, and doctors. The role of active management and passive management are also presented.

The framework of our system is shown in Figure 1. The four critical components in the system are the users, hardware device, cloud service, and doctors. Users first apply our hardware device to collect ECG data; the collection process is displayed simultaneously on a mobile phone. The hardware device sends the data to the cloud service through a mobile phone, and the cloud service sends the automatic ECG analysis results back to the phone. Cardiovascular health management occurs in two ways: (1) actively, by consulting with a doctor; (2) passively, by triggering predefined conditions.

In the subsequent sections, we introduce the data acquisition, automatic ECG analysis, and design of the cardiovascular health management system in detail.

### 2.2. Data Acquisition

As the mobile system works outside a hospital, the main goal of the data acquisition is to guarantee data of a high quality and fidelity while also reducing the size of the recorder.

#### 2.2.1. Hardware Design

**Figure 2:**
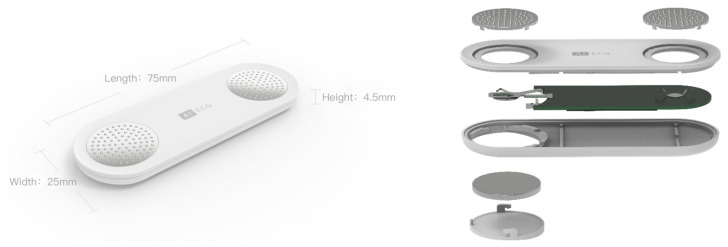
Design of electrocardiogram (ECG) data acquisition hardware.

Our prototype is shown in Figure 2. To acquire ECG data at any time and any location, we designed a small printed circuit board (PCB) for our particular purposes (the middle green board shown on the right side in the figure). Overall, our PCB integrates an ECG collection unit and a Bluetooth low energy (BLE) microcontroller unit. The ECG collection unit further integrates an instrumentation amplifier, two-pole high-pass filtering, and three-pole low-pass filtering. The cut-off frequencies of the filters are 0.5–40 Hz. The sampling frequency of the ECG collection unit is 125 Hz, which is sufficient for a human ECG, according to the Nyquist theorem [16] and the dominant power spectral density of the ECG [17]. In addition, our ECG collection unit can also automatically detect an empty signal when the lead drops. The BLE is used to transmit data from the hardware device to the mobile phone and provides considerably reduced power consumption and cost while maintaining a similar communication range [18]. A button battery powers the PCB.

Two circular metal plates are used as electrodes (also known as “leads”) to connect the body as a circuit between the thumbs. This type of connection is equivalent to Lead I in a standard 12-lead ECG system used in a hospital, which is one of the most significant leads. Each metal plate has numerous small bumps to increase the friction of the thumb, allowing for more stable use of the device and improving the quality of data acquisition.

Finally, we encapsulate the PCB, battery, and electrodes into a small portable hardware device (Figure 2, left). The device weighs approximately 10 g and has a length of 75 mm, width of 25 mm, and height of 4.5 mm. Thus, the device looks similar to a pack of chewing gum and is extremely easy to carry in daily life.

#### 2.2.2. Improved Data Quality

In addition to previously introduced hardware designs to improve the data quality, we also consider the software design.

Prior to the data acquisition, we must ensure that the user’s thumbs are correctly placed on the negative and positive leads. Thus, we designed an AI model to automatically detect whether the thumbs placed on the leads are placed in the correct (0) or reversed (1) way. Our automatic ECG analysis model is similar to a deep neural network, but the final prediction layer is replaced with a sigmoid activation function, which we introduce in Section 2.3.

After the data acquisition, we postprocess the ECG based on digital signal processing [19,20]. We consider two types of distortion: high-frequency noise and low-frequency baseline wandering. High-frequency noise is usually generated by other electrical devices such as electrical watches or irrelevant physiological signals like electromyograms (EMGs). We apply a joint 50-Hz low-pass filter and a 60-Hz notch filter to eliminate high-frequency noise. A low-frequency baseline wandering occurs when physical movement is detected, and it is removed by a 0.67-Hz high-pass filter and moving average filter.

### 2.3. Automatic ECG Analysis with Artificial Intelligence (AI) and Pattern Recognition

#### 2.3.1. Diagnosing an ECG through Deep Neural Network

Deep neural networks (or deep learning methods) have been the most popular AI methods in recent years and have achieved a state-of-the-art performance in many areas, including speech recognition, computer vision, and natural language processing [21]. AI techniques have recently shown significant potential in cardiology [22,23,24,25,26,27] owing to their ability to automatically learn effective features from data without the help of domain experts. When focusing on deep learning methods applying ECG data, various architectures have been proposed for disease detection [15,17,28,29,30,31,32,33,34,35,36,37,38], sleep staging [39,40], and biometric identification [41,42,43,44], among others (see a recent survey in [22]).

However, most of these methods have not attained the stage of practical usage because none have been deployed as a cloud service for cardiovascular health management. Herein, we introduce deep neural networks into ECG diagnosis to facilitate cardiovascular health management.

**Figure 3:**
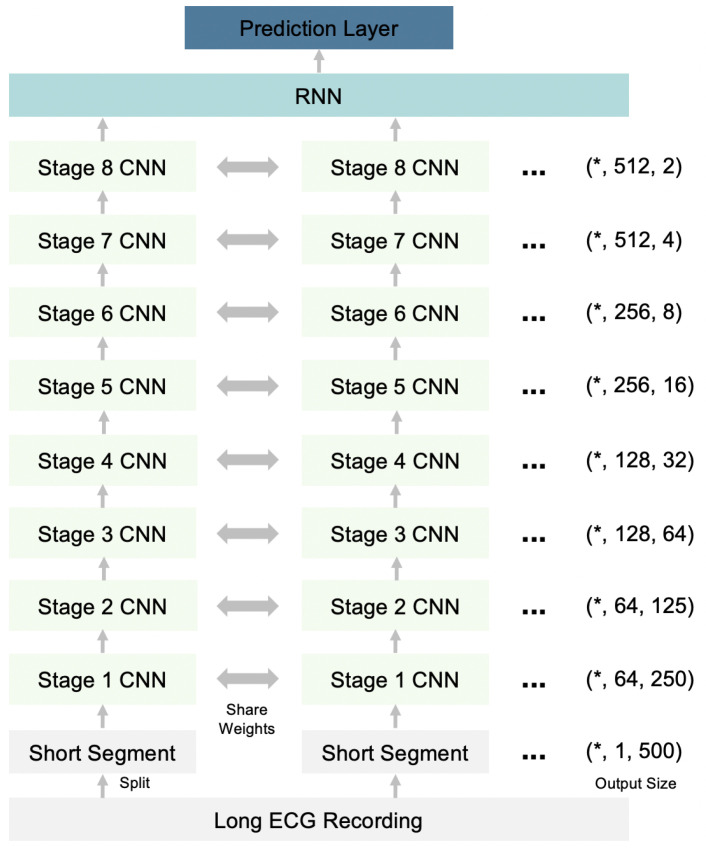
Architecture of the deep neural network for ECG diagnosis. RNN-Recurrent Neural Network; CNN—Convolutional Neural Network. *—User defined batch size.

Our deep neural network is a hybrid of a Convolutional Neural Network (CNN) and a Recurrent Neural Network (RNN), which have shown efficient performance in the modeling of ECG data [22]. As shown in Figure 3, the input-long ECG recording is split into short segments, and each segment is transmitted through 32 layers of stacked one-dimensional convolutional layers to capture local ECG patterns and shifts. The convolutional layers share weights between different segments. These 32 convolutional layers are grouped into eight stages, where each stage is a cascade of four one-dimensional convolutional layers with a kernel size of 16. Every two convolutional layers are residually connected with a shortcut connection [45,46]. In each stage, the first convolutional layer downsamples the input length (the last dimension of the output size in Figure 3) by a factor of 2. Meanwhile, the corresponding shortcut connections also downsample the identity input using a max pooling operation by a factor of 2. The number of filters also increases after every two stages (second dimension of the output size in Figure 3). Before each convolution layer, a nonlinear transformation occurs, which is a combination of batch normalization [47], ReLU activation [48], and a dropout [49]. After eight stages of convolutional layers, the last dimension is reduced by the global average layer. One recurrent layer is then built on top of the convolutional layers to capture long-term variations. The final prediction layer is a fully connected dense layer used to obtain the probabilities of each diagnosis class. The objective of the model is a multilabel learning task because a single ECG recording can have multiple diagnosis results.

Our model supports 20 types of diagnostic items, including sinus rhythm, tachyarrhythmia, and bradyarrhythmia, summarized in Table 1. To train the model, we used the anonymous ECG recordings training set collected from the electrocardiology departments of several tertiary hospitals in China. Each ECG recording lasting from 20 s to several minutes.

#### 2.3.2. Measuring ECG through Pattern Recognition

**Figure 4:**
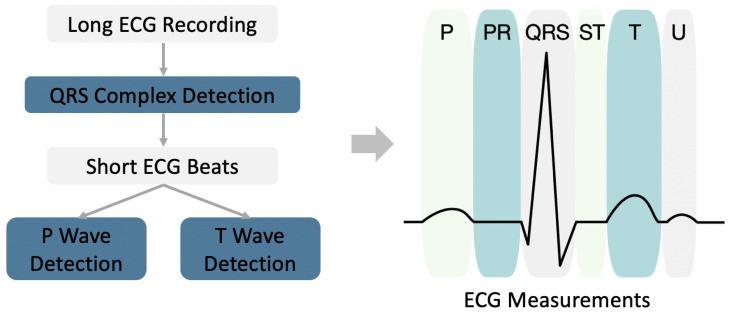
Diagram of ECG measurement based on pattern recognition.

Pattern recognition methods play essential roles in traditional ECG analysis methods. They were developed and accepted by cardiologists during the past few decades. For example, ECG measurements are the most important primary step in the interpretation of an ECG by clinicians for the purpose of diagnosis. Such ECG measurements can describe what an ECG looks like (Figure 4). Although deep learning methods achieve superior performance in ECG diagnoses, they do not perform equally well in ECG measurements. In this study, we also implemented some pattern recognition methods to obtain ECG measurements, which serve as a supplement to deep learning methods.

A diagram of an ECG measurement is shown in Figure 4. For a long ECG recording, the ECG is first split into short beats using QRS complex detection. The P and T waves are then detected based on the beat level. After detecting the onset/peak/offset of the P/QRS/T waves, we can further calculate the following ECG measurements: P wave duration, PR interval, QRS interval, QT interval, QTc interval, T wave duration, atrial rate, and ventricular rate (also known as heart rate).

#### 2.3.3. Deployment as a Cloud Service

We deployed deep learning models and pattern recognition methods on a cloud machine for use as cloud services. We used the Java Client TensorFlow Serving gRPC Application Programming Interface (API). The information transmitted between servers and clients is based on the HTTP protocol. One request of the HTTP includes a HEADER and POST. The HEADER includes authorization information. The authority is a unique token provided by the server. We use Tencent Cloud for our service.

### 2.4. Design of Cardiovascular Health Management

We will now introduce our designed cardiovascular health management system based on previous techniques. Our goal is to provide easy-to-use cardiovascular health management for everyone.

We built a lightweight mobile application based on the WeChat Mini Program, the most popular mobile App in China. This allows users to learn how to use the system at nearly zero cost. We then propose both active and passive methods for health management.

#### 2.4.1. Passive Health Management

For passive health management, the mobile system will automatically detect notable events and alert the user. To achieve this, we first consider the critical value of the indications [50,51,52] and create a new cardiovascular health score. The mapping of diagnostic indications to such scores is shown in Table 2. The score is 100 if no abnormality is detected, and values are subtracted upon detecting corresponding diagnosis items. Subsequently, as advised by cardiologists, we proposed two conditions that require further actions by a doctor: lower than 85 points for two consecutive measurements and lower than 90 points for three measurements, in the most recent five measurements. The heart health score is used as a bridge between outside the hospital and inside the hospital.

#### 2.4.2. Active Health Management

Active health management provides sufficient information based on previous techniques, but it requires users or doctors to initiate health management. Our application can provide a retrospective analysis of historical ECG data commonly used in the medical area. Specifically, our retrospective analysis includes statistics of diagnostic results from a deep learning model, ECG measurements, average beats, heart rate variation (HRV), and a scatterplot of RR intervals. These retrospective analyses are summarized as a unified report and shared with users through WeChat each week. In addition, users can also consult their doctors directly online, accompanied with their reports. Doctors can also estimate the cardiovascular health conditions of the patients more efficiently and take action as quickly as possible.

## 3. Results and Discussion

In this section, we describe the results of methods mentioned earlier, including data-quality improvement (removing high-/low-frequency distortion and reverse lead detection), the efficiency of the cloud service, and the performance of the automatic ECG analysis (deep learning model and pattern recognition method). Finally, we describe a demonstration of health management reports from real-world applications.

### 3.1. Data-Quality Improvement

Data-quality improvement consists of three parts: removing high-frequency noise, low-frequency baseline wandering, and reverse lead detection.

**Figure 5:**
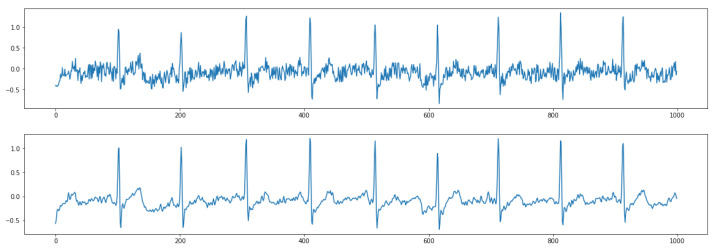
Results of removing high-frequency noise. (**Top**) raw data; (**Bottom**) clean data.

**Figure 6:**
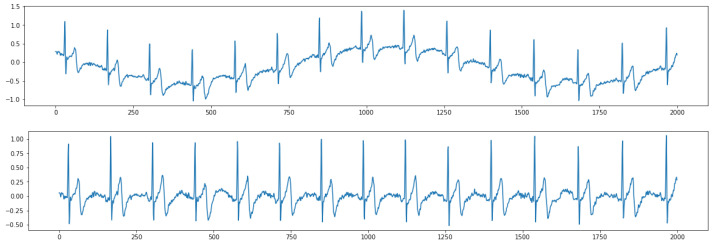
Results of removing low-frequency baseline wandering. (**Top**) raw data; (**Bottom**) clean data.

Figure 5 shows the results of removing high-frequency noise. The top figure shows raw contaminated ECG data with the noise. After postprocessing the ECG data based on a digital signal filter introduced in Section 2.2.2, the output ECG data are smoother without jitters (bottom figure). Figure 6 shows the results of removing low-frequency baseline wandering. The top figure shows raw contaminated ECG data with low-frequency baseline wandering. The bottom figure shows clean data with a stable baseline. Meanwhile, in Figure 5 and Figure 6, we can still clearly recognize a P wave, QRS complex, and T wave in the clean data. Hence, important information is retained to better support the following automatic ECG analysis.

**Figure 7:**
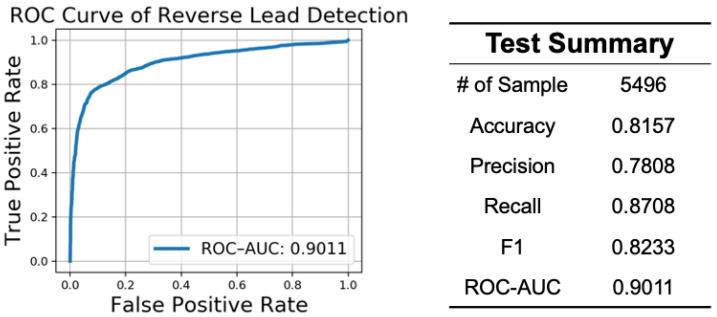
Performance of reverse lead detection. ROC–AUC—Area Under the Receiver Operating Characteristic Curve.

The performance of reverse lead detection is shown in Figure 7. We tested the detector on 5479 ECG recordings from our hardware device, wherein 2748 ECG recordings were correct (0) and 2748 were reversed (1). The figure on the left shows the receiver operating characteristic (ROC) curve, in which the x-axis represents a false positive rate, and the y-axis represents a true positive rate. The ROC–AUC score, which is the area under the ROC curve, is 0.9011. The performance suggests that our reverse lead detector has a good ability to distinguish a correct lead from a reverse lead. The results of other classification evaluations are shown in the right side of the table.

### 3.2. Efficiency

**Figure 8:**
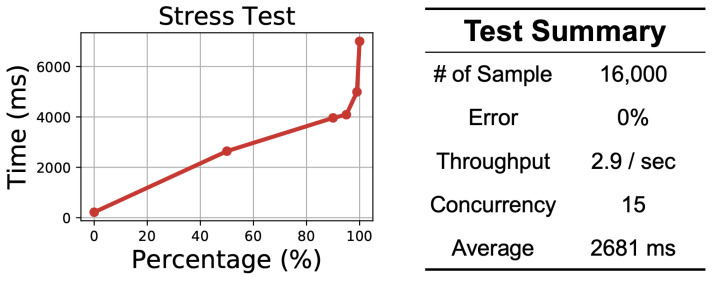
Results of the cloud service efficiency test.

To validate the efficiency of a cloud service, we conducted a stress test using Apache Jmeter [53]. We prepared 16,000 samples of simulated 30-s single-lead ECG recordings and operated the stress test from the local machine to the cloud service. Each cloud server is equipped with a 4-core Intel Xeon Sliver 4110 (2.1 GHz) with 20 GB RAM and a Tesla P4 GPU for model inference. The results are shown in Figure 8. The figure on the left shows the distribution of the process time. We can see that 99% of the requests can be returned within less than 5 s. The table on the right shows the test summary. We can see that the total throughput is 2.9 records per second, which means that our cloud service can analyze approximately 250,000 30-s ECG recordings per day.

### 3.3. Performance of Automatic ECG Analysis

We first tested our deep learning model on a hold-out test set, which was not used to train the model. The test set includes 15,437 anonymous ECG recordings collected from several tertiary hospitals in China. The lead we used is the same lead between limbs, and down-sample a raw ECG to 125 Hz. Each ECG recording was annotated and revised by at least two cardiologists. We extracted the final diagnostic items as ground-truth labels, which covered all of our supported diagnostic items. The results are shown in Table 3. Almost all items achieve higher than 0.95 ROC–AUC scores except Sinus Rhythm, which is a broad group containing various situations. Notably, 17 of them are higher than 0.98. These results suggest that our deep learning model can predict cardiovascular abnormalities from an ECG with extremely high accuracy.

**Figure 9:**
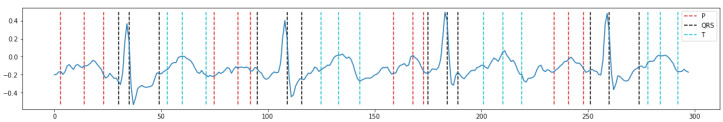
Results of ECG measurements based on pattern recognition. Every three consecutive dashed lines indicate the onset, peak, and offset of the P/QRS/T waves.

We then evaluate the performance of wave detection. As annotating P/QRS/T waves is time-consuming, we conducted an evaluation through a case study. Figure 9 shows a random case of wave detection. Every three consecutive dashed lines indicate the onset, peak, and offset of P/QRS/T waves. This case suggests reasonable boundaries of P/QRS/T waves. More cases can be provided upon request.

### 3.4. Demonstration

**Figure 10:**
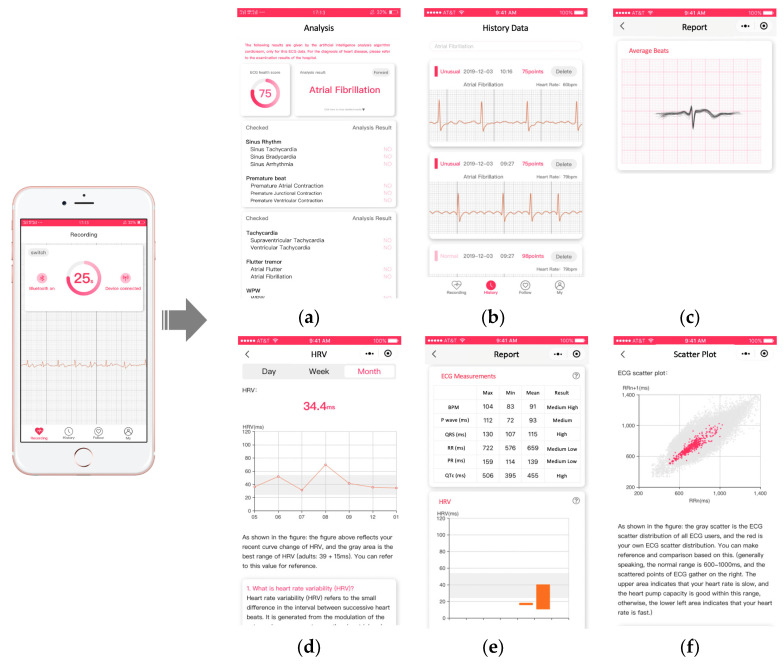
Demonstration of user interfaces based on WeChat Mini Program. (**a**): analysis results; (**b**): history data; (**c**): average beats; (**d**): HRV trend; (**e**): ECG measurements; (**f**)): scatterplot.

Finally, we show some screenshots of our mobile health management WeChat Mini Program. The left-most image in Figure 10 shows the process of ECG collection. The six figures on the right show the analysis results (a), history data (b), average beats (c), HRV trend (d), ECG measurements (e), and scatterplot (f). All interfaces are kept as simple as possible by removing unnecessary contents, aiming to provide clear and easy-to-understand information to ordinary users.

### 3.5. Discussion

This paper proposes a mobile system for cardiovascular health management enhanced with AI. It has achieved initial success in China. The comparison of similar products around the world is shown in Table 4. Overall, our device is smaller and lighter, while supporting more diagnostic items. Although this devices does not use a rechargeable lithium battery, the working power consumption is only 50 uA, while sleeping power consumption is 0.5 uA. It is estimated to be used for one month with one button cell (CR2016). Our next generation product equipped with a rechargeable lithium battery is on the way. Besides, as shown in Figure 1, the AI algorithm, databases, and health management are provided by cloud service, so the requirement of memory storage is very little.

Our system has several limitations. First, our hardware device is not wearable; therefore, it cannot collect ECG data continuously. Thus, some paroxysmal cardiovascular diseases might be overlooked. The second limitation is the opaque mechanism of deep learning models. Our model contains millions of trainable parameters and nonlinear connections. It is complicated and cannot be fully understood by doctors. To alleviate this, we also designed attention mechanisms [54] in convolutional and recurrent layers. This allows users to determine the model’s mechanism by visualizing the attention weights. However, considerable time is required to fully comprehend the deep learning models.

In the future, we plan to promote both the hardware and software techniques. For the hardware, we will design a more convenient ECG data acquisition tool, for example, a comfortable wearable device that can be placed on the skin throughout the day. The ECG data collection can thus last for the entire day, allowing more paroxysmal cardiovascular diseases to be detected. For the software, we plan to implement other advanced AI techniques based on machine learning and data mining research. For example, we plan to use ensemble learning to further improve the model’s performance. We also plan to implement semisupervised learning for better use of unlabeled data.

## 4. Conclusions

In this paper, we proposed a mobile system for cardiovascular health management. We developed a portable system with high-quality data acquisition for use in a natural environment. We improved the mobile system using advanced AI algorithms to provide accurate ECG diagnostic results. The user interface of the system application is easy-to-use for the elderly population. Hence, ordinary people can easily apply it as a powerful tool to manage their cardiovascular health.

## Figures and Tables

**Table 1:** Items of ECG diagnosis supported by our deep neural network.

Group	Subgroup	Name	Abbr.
Sinus Rhythm	Sinus Rhythm	Sinus Rhythm	SN
	Sinus Abnormality	Sinus Arrhythmia	SNA
		Sinus Tachycardia	SNT
		Sinus Bradycardia	SNB
Tachyarrhythmia	Premature Beat	Premature Ventricular Contraction	PVC
		Premature Junctional Contraction	PJC
		Premature Atrial Contraction	PAC
	Tachycardia	Ventricular Tachycardia	VT
		Supraventricular Tachycardia	SVT
	Flutter and Fibrillation	Atrial Flutter	AFL
		Atrial Fibrillation	AF
	Pre-excitation	Wolff–Parkinson–White Syndrome	WPW
Bradyarrhythmia	Escape Beat	Ventricular Escape	VE
		Atrial Escape	AE
		Junctional Escape	JE
	Atrioventricular Block	First Degree Atrioventricular Block	AVBI
		Second Degree Atrioventricular Block	AVBII
		Third Degree Atrioventricular Block	AVBIII
	Intraventricular Block	Left Bundle Branch Block	LBBB
		Right Bundle Branch Block	RBBB

**Table 2:** Categories and critical values of diagnostic items.

Category	Diagnosis Items (Critical Value)
No Risk	SN (0)
Medium-low Risk	SNA (2), SNT (5), SNB (5)
Medium Risk	LBBB (9), PVC (12), PJC (12), PAC (12), RBBB (15)
Medium-high Risk	WPW (16), VE (16), AE (16), JE (16), AVBI (16), AFL (25), AF (25), AVBII (25)
High Risk	VT (50), SVT (50), AVBIII (50)

**Table 3:** Performance of our deep neural network on the test set.

Abbr.	Accuracy	Precision	Recall	F1	ROC–AUC
SN	0.9862	0.9881	0.9978	0.9929	0.8948
SNA	0.9553	0.4977	0.6479	0.5629	0.9564
SNT	0.9888	0.8800	0.8761	0.8780	0.9948
SNB	0.9842	0.8685	0.9413	0.9034	0.9968
PVC	0.9946	0.8659	0.8505	0.8582	0.9852
PJC	0.9977	0.1429	0.5556	0.2273	0.9973
PAC	0.9861	0.6771	0.8320	0.7466	0.9855
VT	0.9997	0.8333	0.5556	0.6667	0.9916
SVT	0.9975	0.4865	0.5000	0.4932	0.9894
AFL	0.9984	0.7407	0.5556	0.6349	0.9967
AF	0.9974	0.8941	0.8786	0.8863	0.9989
WPW	0.9989	0.8333	0.6944	0.7576	0.9954
VE	0.9990	0.2105	1.0000	0.3478	0.9992
AE	0.9980	0.0938	0.7500	0.1667	0.9968
JE	0.9988	0.6190	0.5652	0.5909	0.9967
AVBI	0.9963	0.6838	0.8163	0.7442	0.9973
AVBII	0.9993	0.7778	0.4667	0.5833	0.9967
AVBIII	0.9997	0.8750	0.7000	0.7778	0.9974
LBBB	0.9984	0.7705	0.8246	0.7966	0.9837
RBBB	0.9911	0.8776	0.6804	0.7665	0.9616

**Table 4:** Comparison of similar products.

Supplier	Ours	iRhythm	Apple	Qardio
Model	E-HA03	Zio SR Patch	Apple Watch	QardioCore
User Interface	WeChat Mini-Program	/	iOS	iOS
Lead	Single-lead	Single-lead	Single-lead	3-leads
Carrying	Portable	Patch	Wrist	Chest
Diagnostic items	SN, SNA, SNT, SNB, PVC, PJC, PAC, VT, SVT, AFL, AF, WPW, VE, AE, JE, AVBI, AVBII, AVBIII, LBBB, RBBB	SN, AF, AFL, VT, SVT, VE, PVC, PAUSE, AVB	SN, AF	SN, AF, SNB, SNT, PAUSE
Transmission	Bluetooth	/	/	Bluetooth
Battery	Button Cell	Lithium	Lithium	Lithium
Size (mm)	75 × 26 × 6	110 × 60 × 20	/	185 × 87 × 9
Weight	12 g	34 g	56 g	130 g

## Data Availability

The dataset used to support the findings of this study are available on request from the corresponding author.

## Abbreviations

The following abbreviations are used in this manuscript:

AI	Artificial Intelligence
AE	Atrial Escape
AF	Atrial Fibrillation
AFL	Atrial Flutter
AVBI	First Degree Atrioventricular Block
AVBII	Second Degree Atrioventricular Block
AVBIII	Third Degree Atrioventricular Block
BLE	Bluetooth low energy
CNN	Convolutional Neural Network
ECGs	Electrocardiograms
EEGs	Electroencephalograms
EHRs	Electronic health records
EMGs	Electromyograms
HRV	Heart rate variation
IoT	Internet of Things
JE	Junctional Escape
LBBB	Left Bundle Branch Block
PAC	Premature Atrial Contraction
PCB	Printed circuit board
PJC	Premature Junctional Contraction
PPG	Photoplethysmogram
PVC	Premature Ventricular Contraction
RBBB	Right Bundle Branch Block
RNN	Recurrent Neural Network
ROC	Receiver operating characteristic
ROC–AUC	Area Under the Receiver Operating Characteristic Curve
SN	Sinus Rhythm
SNA	Sinus Arrhythmia
SNB	Sinus Bradycardia
SNT	Sinus Tachycardia
SVT	Supraventricular Tachycardia
VE	Ventricular Escape
VT	Ventricular Tachycardia
WPW	Wolff-Parkinson-White Syndrome
